# Effect of Alkaloids Isolated from* Phyllodium pulchellum* on Monoamine Levels and Monoamine Oxidase Activity in Rat Brain

**DOI:** 10.1155/2016/6826175

**Published:** 2016-04-18

**Authors:** Lu Cai, Chao Wang, Xiao-kui Huo, Pei-pei Dong, Bao-jing Zhang, Hou-Li Zhang, Shan-shan Huang, Bo Zhang, Sheng-ming Yu, Ming Zhong, Xiao-Chi Ma

**Affiliations:** ^1^College of Pharmacy, Dalian Medical University, Dalian 116044, China; ^2^Department of Neurosurgery, The Second Affiliated Hospital, Dalian Medical University, Dalian 116044, China; ^3^Institute of Nationality Medicine in Guangxi, Nanning 530001, China

## Abstract

*Phyllodium pulchellum* (*P. pulchellum*) is a folk medicine with a significant number of bioactivities. The aim of this study was to investigate the effects displayed by alkaloids fractions, isolated from the roots of* P. pulchellum*, on neurotransmitters monoamine levels and on monoamine oxidase (MAO) activity. Six alkaloids, which had indolealkylamine or *β*-carboline skeleton, were obtained by chromatographic technologies and identified by spectroscopic methods such as NMR and MS. After treatment with alkaloids of* P. pulchellum*, the reduction of DA levels (54.55%) and 5-HT levels (35.01%) in rat brain was observed by HPLC-FLD. The effect of alkaloids on the monoamines metabolism was mainly related to MAO inhibition, characterized by IC_50_ values of 37.35 ± 6.41 and 126.53 ± 5.39 *μ*g/mL for MAO-A and MAO-B, respectively. The acute toxicity indicated that* P. pulchellum* extract was nontoxic.

## 1. Introduction

*Phyllodium pulchellum* (*P. pulchellum*) Desv. (Leguminosae) is a folk medicine distributed in the southern parts of China, with a significant number of bioactivities especially for the central nervous system (CNS).* P. pulchellum* exhibited hypothermia and mild analgesic effects on rheumatoid arthritis. Recently, many investigations suggested that the ethanol extract of* P. pulchellum* had significant bioactivity* in vivo* system of liver fibrosis [1, 2].* P. pulchellum* is widely used in traditional medicine with no literature evidence substantiating its safety. To optimize their safe use, it would be urgent to study the safety and the chemical foundation.

Previous research had shown that chemical constituents of* P. pulchellum* included alkaloids, alcohols, and phenols. In total alkaloids part, indolealkylamine (IAA) and *β*-carboline type alkaloids were its main constituents [3–5]. To the best of our knowledge, these alkaloids are regarded as the promising plant-derived compounds to treat CNS illnesses, due to their unique complex nitrogen-containing structures to interact with diverse neuronal and molecular targets [6]. IAA drugs were 5-hydroxytryptamine (5-HT or serotonin) analogs that mainly act on a variety of 5-HT receptors, serotonin transporter, or even MAO enzyme that were highly favorable molecular targets for treating depression, anxiety, schizophrenia, and other psychiatric disturbances [7, 8]. Additionally, *β*-carbolines were naturally occurring alkaloids that exhibited a wide range of psychopharmacological effects due to their binding to benzodiazepine, imidazoline, serotonin, and opiate receptors as well as monoamine oxidase (MAO) activity inhibition [9, 10].

Dopamine (DA) and serotonin (5-HT) play a major role as neurotransmitters in the control and regulation of the central nervous system. DA is one of the most important excitatory neurotransmitters, involved in a variety of behaviors and brain functions, such as motor activity, cognition, emotion, positive reinforcement, food intake, and endocrine regulation [11]. Changes on DA transmission are associated with Parkinson's disease and schizophrenia [12]. 5-HT, as a conventional neurotransmitter, is involved in the regulation of mood, sleep, memory, learning, and sexual behavior as a crucial fine-tuner of normal and pathological processes [13]. Alterations in 5-HT transmission are related to some neurological and psychiatric illness including migraine, hallucinations, anxiety, and depression [14].

Monoamine oxidases (MAOs) are mitochondrial bound isoenzymes which catalyze the oxidative deamination of monoamine neurotransmitters, including 5-HT, histamine, and catecholamines (dopamine, noradrenaline, and adrenaline). MAO is classified into two types (**A** and** B**), according to their sensitivity towards specificity substrates and inhibitors. MAO-A shows a higher affinity for 5-HT and noradrenaline and is selectively inhibited by clorgyline, whereas MAO-B preferentially deaminates phenylethylamine and benzylamine and is selectively inhibited by l-deprenyl or pargyline. Dopamine is oxidized by both forms of the enzyme in most species [15]. MAO-A inhibitors have proven to be effective in the pharmacological treatment of depression, and further developments have provided reversible inhibitors of MAO-A, which offer antidepressant activity without the serious side effects of the earlier inhibitors. On the other hand, selective inhibitors of MAO-B have found a therapeutic role in the treatment of Parkinson's disease [16].

Considering the presence of indolealkylamine and *β*-carboline alkaloids compounds in* P. pulchellum* and the previous effects described for these alkaloids on the CNS, it becomes relevant to investigate the effects of fractions of* P. pulchellum* on monoamines metabolites and MAO activity. In order to give vital guidance to uses and further developments of* P. pulchellum*, the investigation regarding chemical constituents of* P. pulchellum* alkaloid and its influences of monoamine levels and monoamine oxidases (MAO-A and -B) was carried out in the present paper.

## 2. Results and Discussion

### 2.1. Acute Toxicity

The results of the acute toxicity were shown in Table 1. There was a regular dose-dependent increase in mortality and decrease in mortality latency in both sexes of mice after the administration of* P. pulchellum* extract. The first mouse died between 72 and 120 h after injection of the 8000 mg/kg dose of the extract, and the maximum frequency of death occurred at 20000 mg/kg. The no-observed–adverse-effect (NOAEL) dose for the extract was 6000 mg/kg, the maximum tolerated dose (MTD: highest dose at which the mice recovered completely) was assumed to be between 6000 mg and 8000 mg/kg, and the single dose LD_50_ was 11300 mg/kg (95% confidence limit: 9762–13075 mg/kg). The symptom of weight loss was observed during the later stages of the experiment. The histological analysis showed an absence of alterations in all organs examined (results were not shown).

*P. pulchellum* is widely used traditionally in the southern parts of China with no literature evidence substantiating its safety, so it is necessary to evaluate the toxicity of this medicinal herb. In the present study, the LD_50_ of* P. pulchellum* extract was 11300 mg/kg, based on the classification of Loomis and Hayes [17], namely, that substances with LD_50_ between 5000 and 15000 mg/kg bodyweight are regarded as being practically nontoxic. However, some mild adverse effects such as dizziness, trembling, crouching, and sluggishness were observed, and the effect was reversible within 30 min and vanished after 1 hr.

### 2.2. Chemical Constituents

**Figure 1:**
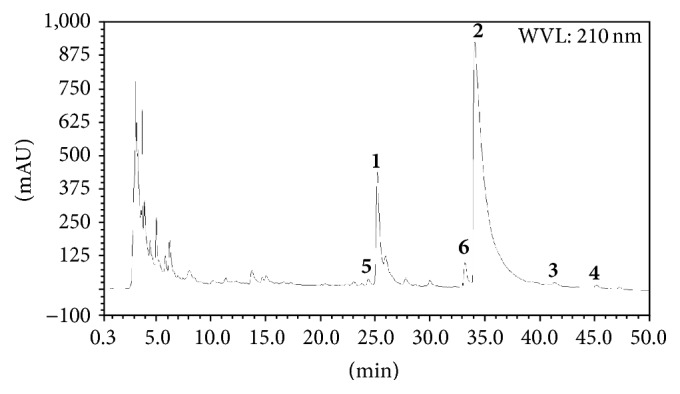
Chromatogram of total alkaloids of* Phyllodium pulchellum* with six alkaloids (**1**–**6**) indicated (210 nm).

The total alkaloids were obtained as the CHCl_3_ extracted materials from the hydrochloric acid water extract of the roots of* P. pulchellum* with the content of 0.12%. It was also analyzed by HPLC subjected to a RP C18 column at 30°C with DAD detection. The mobile phase was comprised of water (solvent A) and acetonitrile (solvent B) both acidified with 0.03% CF_3_COOH using a gradient manner: 5% B– 35% B for 90 min, at 0.8 mL/min. HPLC chromatograms of total alkaloids were recorded with UV detection at 210 nm, as shown in Figure 1.

**Figure 2:**
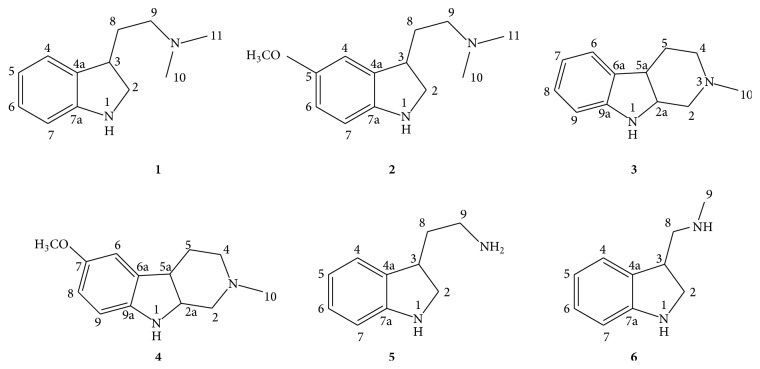
The alkaloids** 1**–**6** isolated from the roots of* P. pulchellum*.

Totally, six alkaloids were obtained by various chromatography techniques (Figure 2). They were elucidated as N,N-dimethyltryptamine (**1**), 5-methoxy-N,N-dimethyltryptamine (**2**), N-methyltetrahydrocarboline (**3**), 7-methoxy-N-methyltetrahydrocarboline (**4**), tryptamine (**5**), and N-methyl-3-indoylmethanamine (**6**) from their spectroscopic data upon comparisons with values reported in the literature [18]. According to the structure characteristics, six alkaloids can be divided into two different structure styles: compounds** 1**,** 2**,** 5**, and** 6** belonged to indolealkylamine and compounds** 3** and** 4** were *β*-carboline alkaloids.

### 2.3. HPLC-FLD Analysis

In the present study, the monoamine levels of DA and 5-HT and their main metabolites (DOPAC and 5-HIAA) were quantified by HPLC-FLD in brain of rats after treatment with alkaloids of* P. pulchellum*. The concentrations of DA, DOPA, 5-HT, and 5-HIAA in experimental group were significantly different from the control group (Table 2). In dopaminergic system, a decrease of 54.55% in DA levels and a decrease in the levels of metabolites DOPAC (35.54%) were observed. The ratios of metabolites to monoamines were evaluated to estimate the activity of brain monoamine metabolism [19]. There was a significant decrease in DA concentration associated with an increased DOPAC/DA ratio of this monoamine after the administration of* P. pulchellum*. These data indicated that* P. pulchellum* may cause alterations in DA and metabolites levels in rat brain.

In serotonin system, there was a significant reduction in the 5-HT levels (35.01%) following the decreased levels of 5-HIAA (53.45%). The ratio 5-HIAA/5-HT is frequently employed as a serotonin metabolism indicator, since it establishes the 5-HT consumption and the formation of its metabolites product. In this assay, when rats received* P. pulchellum* alkaloids, the serotonin turnover ratio (5-HIAA/5-HT) was decreased from 0.94 to 0.67, which indicated that alkaloids attenuated the monoamines metabolism in this brain area.

The main chemical constituents of* P. pulchellum* were alkaloids which had indolealkylamine skeleton and 5-methoxy-N,N-dimethyltryptamine (5-MeO-DMT) as major substances. The indolealkylamine alkaloids showed a relatively high oil/water partition coefficient, suggesting that the alkaloids may easily penetrate various lipoprotein barriers including the blood brain barrier. As shown previously [20, 21], 5-MeO-DMT significantly accumulates in many organs (e.g., liver, kidney, and brain) in different animal models. The brain concentration of 5-MeO-DMT is about 1.7-fold higher than that in blood at 45 min after administration, and the drug is widely distributed in different rat brain regions including cortex, thalamus, hippocampus, basal ganglia, medulla, pons, and cerebellum.

The alkaloids from* P. pulchellum* produced a short-lived decrease in locomotor activity and investigatory behavior in rats after oral administration. The hypoactivity effect could be attributed to the presence of indolealkylamine (IAA) alkaloids in* P. pulchellum*, because IAA compounds were 5-HT analogs that mainly act on a variety of 5-HT receptors or serotonin transporters, which have been used in social and religious cultures throughout history [22].

### 2.4. MAO

**Figure 3:**
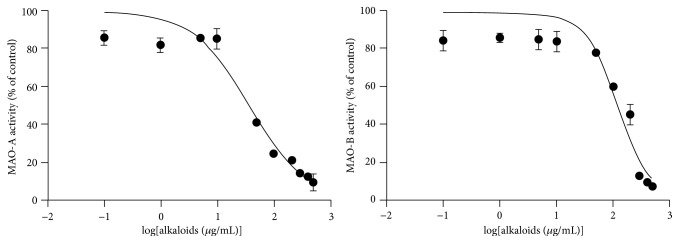
Effects displayed by* P. pulchellum* on MAO-A and MAO-B activity.* P. pulchellum* alkaloid was tested in ten different concentrations ranging from 0.1 to 500 *μ*g/mL. The degree of inhibition IC_50_ was assessed by a sigmoidal dose-response curve. Each point represents the mean ± SMD of the sigmoidal regression for three independent determinations.

The effect of alkaloids isolated from* P. pulchellum* on MAO was evaluated in mitochondrial fractions from rat brain. As shown in Figure 3,* P. pulchellum* alkaloids significantly inhibited both MAO-A and MAO-B activity in a concentration-dependent pattern. The IC_50_ values calculated for alkaloids (37.35 ± 6.41 and 126.53 ± 5.39 *μ*g/mL for MAO-A and MAO-B, resp.) indicated high potency against MAO-A. Additionally, regarding the activity against MAO-A, the tested extracts displayed maximum inhibition above 85% in the highest concentrations evaluated.

The inhibition observed on MAO activity is in agreement with the reduction in DOPAC levels in rat brain, since DA is a nonselective substrate for MAO-A and MAO-B, being converted to DOPAC by both enzymes. The selective inhibition on MAO-A is in agreement with the reduction in 5-HIAA levels, since 5-HT is a selective substrate for MAO-A.

In addition to indolealkylamine alkaloids, we also isolate and identify the *β*-carboline alkaloids: compound** 3** (N-methyltetrahydrocarboline) and compound** 4** (7-methoxy-N-methyltetrahydrocarboline). So far, studies of the activity of the compounds have not been reported in the literature; however, reports were found for chemically related alkaloids such as harmine, harmaline, and tetrahydroharmine, which are potent and reversible inhibitors of MAO [23, 24]. Therefore, the alkaloids present in the aqueous extract from* P. pulchellum* could play a role in the MAO inhibition.

In conclusion, the present study demonstrates that aqueous exact of the root of* P. pulchellum* was nontoxic. The main chemical constituents were alkaloids which had indolealkylamine or carboline skeleton and 5-methoxy-N,N-dimethyltryptamine and N,N-dimethyltryptamine were major substances. The alkaloids seem to act on 5-HT and DA systems in rat brain, affecting the monoamines metabolism and MAO activity.

## 3. Material and Methods

### 3.1. Chemicals

4-Hydroxyquinoline, kynuramine dihydrobromide, selegiline, and clorgyline were obtained from Sigma (St. Louis, MO, USA). All other reagents were of analytical grade.

### 3.2. Plant Material

The roots of* P. pulchellum* were collected in Gongcheng, Guangxi, China, in August 2012 and identified by Chief Physician Bin Dai from the Institute of Nationality Medicine in Guangxi. A voucher specimen has been deposited at the Herbarium of Institute of Nationality Medicine in Guangxi (S-1082).

### 3.3. Animals and Treatment

Wistar rats (200 ± 20 g) and mice (20 ± 2 g) were purchased from the Laboratory Animal Center of Dalian Medical University. Before the experiments, the rats were allowed one-week acclimation period in the animal quarters under air conditioning (22°  ±  1°C, humidity 50–60%) and an automatically controlled photoperiod of 12 h light daily, fed with standard rodent chow and tap water* ad libitum*. The experimental procedures were carried out in accordance with the National Institutes of Health Guide for the Care and Use of Laboratory Animals and were approved by the Animal Care and Use Committee of the Dalian Medical University. After acclimatization, the rats were randomly divided into two groups (*n* = 8). The alkaloids isolated were dissolved in distilled water for oral administration in treated groups at the dosages of 20 mg/kg; the control animals received an equivalent volume of the vehicle. Half an hour after administration, animals were sacrificed by decapitation and brains were immediately removed, washed in ice-cold isolation medium, and stored at −70°C until analyzed.

### 3.4. Acute Toxicity

Acute toxicity was tested using a variation of the method described by Litchfield Jr. and Wilcoxon in [25]. Mice were randomly divided into six groups with five female mice and five male mice in each group. The* P. pulchellum* sample was extracted by decoction and administered by gavage (p.o.) at single doses of 6000, 8000, 11000, 15000, and 20000 mg/kg body weight, while normal saline equivalent to the highest volume of the extract given was administered to the control group. The general behavior of the mice was continuously monitored for 1 h after dosing, periodically during the first 24 h (with special attention given during the first 4 h) [26]. The mice were further observed for up to 14 days after treatment and any signs of toxicity and deaths were recorded. At 15 days, the mice were killed and histological analysis was made of the following organs: heart, kidney, lung, liver, spleen, duodenum, pancreatic, bladder, brain, and genitals.

### 3.5. Extraction and Isolation

The air-dried and powdered roots (10 kg) of* P. pulchellum* were ultrasonically extracted with hydrochloric acid water solution (pH 2-3) (100 Hz × 1 h × 3). After the dregs were filtrated, the aqueous solution was subjected to strongly acidic cation-exchange resin column chromatography and eluted with water till neutrality (pH 7). When the resin was treated with ammonia water, it was extracted by 95% ethanol. After evaporation of ethanol* in vacuo*, the aqueous residue of the ethanolic extract was diluted with water and then partitioned with CHCl_3_. The CHCl_3_ extract (12 g) was obtained as the total alkaloids and subjected to column chromatography on silica gel with petroleum ether-acetone (100 : 1-1 : 1) to afford five fractions (F1–F5). Subfraction F2 (2.3 g) was further isolated on RP-18 column and finally purified by preparative HPLC (5 mL/min, 210 nm, CH_3_CN-H_2_O-CF_3_COOH (10 : 90 : 0.03, v/v/v)) to give compounds** 1** (3.4 mg, Rf 20.8 min) and** 3** (8.5 mg, Rf 22.8 min). Compounds** 2** (8.5 mg, Rf 16.5 min) and** 4** (8.5 mg, Rf 17.4 min) were purified from subfraction Fr3 by pre-HPLC (5 mL/min, 210 nm, CH_3_CN-H_2_O-CF_3_COOH (12 : 88 : 0.03, v/v/v)), and compounds** 5** (8.5 mg, Rf 18.4 min) and** 6** (8.5 mg, Rf 20.2 min) were isolated form Fr 4by pre-HPLC (5 mL/min, 210 nm, CH_3_CN-H_2_O-CF_3_COOH (8 : 92 : 0.03, v/v/v)).

### 3.6. HPLC-FLD Analysis

Detections of the levels of DOPA, DA, 5-HT, and 5-HIAA in brain tissues of mice were carried out by HPLC-FLD [27]. The brain tissue was put into chilled tubes, homogenized in 0.8 *μ*L perchloric acid (0.1 M) on ice. The homogenates were centrifuged at 11,000 ×g for 20 min and 500 *μ*L of the supernatant was ultrafiltrated for another 20 min. The instrument parameters used for HPLC-FLD are as follows: Waters chromatographic system, Diamonsil ODS column (4.6 mm × 250 mm), mobile phase: methanol-buffer (buffer: 0.07 mol NaH_2_PO_4_, 10 mmol sodium octanesulfonate, pH 3.5); flow rate: 1.0 mL/min; injection volume: 20 *μ*L; column temperature: 30°C. Excitation and emission of the fluorescence detector were set to 280 and 315 nm, respectively. The area of peak amplitude was detected and the level of detecting index calculated by a standard curve. The tissue levels of monoamine were expressed in terms of nanograms per gram of tissue. Student's *t*-test was employed for comparisons between control and treated groups. The results were expressed as mean ± SEM (standard error of the mean). When the *P* value was <0.05 (*∗*), the difference was considered significant.

### 3.7. Preparation of Brain Mitochondria

Wistar rats were killed by decapitation. Brains tissues were immediately removed and were mechanically homogenized in a Potter-Elvehjem tissue grinder in 10 vol of the ice-cold sodium phosphate buffer (200 mM, pH 7.4) containing 320 mM sucrose. Then, the homogenate was centrifuged at 3000 ×g for 10 min at 4°C to remove nuclei and cell debris. The mitochondrial fraction was obtained by further centrifugation at 15,000 ×g for 30 min at 4°C and resuspended in PBS buffer (pH 7.4). Protein concentration was determined by the Lowry method [28].

### 3.8. MAO Inhibition Assay

Monoamine oxidase inhibition assays were carried out with fluorescence based method (end-point lecture), as previously described [29, 30]. The substrate used for the assay was kynuramine, which is nonfluorescent until it undergoes oxidative deamination by MAO resulting in the fluorescent metabolite 4-hydroxyquinoline. Product formation was quantified by comparing the fluorescence emission of the samples to that of known amounts of authentic metabolite 4-hydroxyquinoline. Reactions were carried out in black polystyrene 96-well microtiter plates in a final volume of 200 *μ*L. For the MAO-A inhibition assays, the wells containing 120 *μ*L of PBS (pH 7.4), 5 *μ*L of pargyline 10 *μ*M (to get a final concentration of 250 nM), 25 *μ*L of the sample solution prepared in PBS and DMSO (to get a final concentration of 1% DMSO), and 40 *μ*L of the mitochondrial suspension (to get a final protein concentration of 0.1 mg/mL) were preincubated at 37°C for 30 min. The MAO-B inhibition assays were performed in the same way as that of the MAO-A incubations, except by the use of 5 *μ*L of clorgyline (10 *μ*M) to replace the pargyline solution. The assay was started by addition of 10 *μ*L kynuramine (final concentration of 22 *μ*M) and was stopped by addition of 25 *μ*L of 6 M NaOH solution 30 min later. The fluorescence intensity was detected with excitation at 315 nm and emission at 380 nm using a fluorescence spectrometer. Alkaloids were tested in ten different concentrations ranging from 0.1 to 500 *μ*g/mL. Data analysis was performed with GraphPad Prism 5.0 software. The degree of inhibition IC_50_ was assessed by a sigmoidal dose-response curve.

## 4. Conclusion

The alkaloids were main chemical constituents of aqueous exact of the root of* Phyllodium pulchellum*. Structure analyses indicated that the alkaloids have indolealkylamine or carboline skeleton and 5-methoxy-N,N-dimethyltryptamine and N,N-dimethyltryptamine were major substances. The acute toxicity indicated that the alkaloids extract was nontoxic. The alkaloids seem to act on 5-HT and DA systems in rat brain, affecting the monoamines metabolism and MAO activity.

## Figures and Tables

**Table 1:** Effects of *P. pulchellum* alkaloids administered p.o. in mice.

Dose (mg/kg)	Obituary/total animals	Mortality (%)	Mortality latency (h)	LD_50_ (mg/kg)
6000	0/10	0	—	11300
8000	2/10	20	>72, <120	
11000	5/10	50	>24, <48	
15000	7/10	70	>24, <48	
20000	10/10	100	>2, <24	

**Table 2:** Effects on monoamine neurotransmitters and their metabolites of the rat brain after administration of *P. pulchellum* alkaloids (20 mg/kg).

	Control (*n* = 8) (ng/g tissue)	Treated (*n* = 8) (ng/g tissue)
5-HT (35.01%)	50.53 ± 7.53	32.84 ± 5.21^**∗**^
5-HIAA (53.45%)	47.56 ± 6.13	22.14 ± 6.48^**∗∗**^
Dopamine (54.55%)	69.20 ± 12.51	31.45 ± 16.61^**∗∗**^
DOPAC (35.54%)	168.45 ± 18.63	108.58 ± 9.21^**∗∗**^

Results are mean ± SEM (standard error of the mean). Statistical significance:  ^*∗*^ *P* < 0.05 and ^*∗∗*^ *P* < 0.01 compared with control (Student's *t*-test).

## References

[1] Wang C., Zhong M., Zhang B. (2014). Chemical constituents against hepatic fibrosis from *Phyllodium pulchellum* roots. *China Journal of Chinese Materia Medica*.

[2] Huang J., Zhong M., Yu S. (2013). Effects of total alkaloids from *Phyllodium pulchellum* on proliferation of human hepatic stellate cells and collagen, cytokines related to hepatic fibrosis. *Chinese Journal of Experimental Traditional Medical Formulae*.

[3] Ghosal S., Banerjee S. K., Bhattacharya S. K., Sanyal A. K. (1972). Chemical and pharmacological evaluation of *Desmodium pulchellum*. *Planta Medica*.

[4] Shen C.-C., Wang S.-T., Tsai S.-Y., Yang H.-C., Shieh B.-J., Chen C.-C. (2005). Cinnamylphenols from *Phyllodium pulchellum*. *Journal of Natural Products*.

[5] Zong Y., Zhong M., Li D.-M. (2014). Phenolic constituents from the roots of *Phyllodium pulchellum*. *Journal of Asian Natural Products Research*.

[6] Konrath E. L., Passos C. D. S., Klein-Júnior L. C., Henriques A. T. (2013). Alkaloids as a source of potential anticholinesterase inhibitors for the treatment of Alzheimer's disease. *Journal of Pharmacy and Pharmacology*.

[7] Roth B. L., Hanizavareh S. M., Blum A. E. (2004). Serotonin receptors represent highly favorable molecular targets for cognitive enhancement in schizophrenia and other disorders. *Psychopharmacology*.

[8] Yu A.-M. (2008). Indolealkylamines: biotransformations and potential drug-drug interactions. *AAPS Journal*.

[9] Miralles A., Esteban S., Sastre-Coll A., Moranta D., Asensio V. J., García-Sevilla J. A. (2005). High-affinity binding of *β*-carbolines to imidazoline I_2B_ receptors and MAO-A in rat tissues: Norharman blocks the effect of morphine withdrawal on DOPA/noradrenaline synthesis in the brain. *European Journal of Pharmacology*.

[10] Herraiz T., Chaparro C. (2005). Human monoamine oxidase is inhibited by tobacco smoke: *β*-carboline alkaloids act as potent and reversible inhibitors. *Biochemical and Biophysical Research Communications*.

[11] Baptista T., Lacruz A., Pàez X., Hernàndez L., Beaulieu S. (2002). The antipsychotic drug sulpiride does not affect bodyweight in male rats. Is insulin resistance involved?. *European Journal of Pharmacology*.

[12] Verheij M. M., Cools A. R. (2008). Twenty years of dopamine research: individual differences in the response of accumbal dopamine to environmental and pharmacological challenges. *European Journal of Pharmacology*.

[13] Bjorvatn B., Grønli J., Hamre F. (2002). Effects of sleep deprivation on extracellular serotonin in hippocampus and frontal cortex of the rat. *Neuroscience*.

[14] Hou C., Jia F., Liu Y., Li L. (2006). CSF serotonin, 5-hydroxyindolacetic acid and neuropeptide Y levels in severe major depressive disorder. *Brain Research*.

[15] Abell C. W., Kwan S.-W. (2000). Molecular characterization of monoamine oxidases A and B. *Progress in Nucleic Acid Research and Molecular Biology*.

[16] Youdim M. B. H., Bakhle Y. S. (2006). Monoamine oxidase: isoforms and inhibitors in Parkinson's disease and depressive illness. *British Journal of Pharmacology*.

[17] Loomis T., Hayes A. (1996). *Loomis's Essentials of Toxicology*.

[18] Babin F., Huynh-Dinh T. (1987). Chemical and enzymatic oxidative coupling of 5-hydroxy-N,N-dimethyltryptamine with amines. *Journal of Medicinal Chemistry*.

[19] Farias F. M., Passos C. S., Arbo M. D. (2012). Strictosidinic acid, isolated from Psychotria myriantha Mull. Arg. (Rubiaceae), decreases serotonin levels in rat hippocampus. *Fitoterapia*.

[20] Sitaram B. R., Lockett L., Talomsin R., Blackman G. L., McLeod W. R. (1987). *In vivo* metabolism of 5-methoxy-*N, N*-dimethyltryptamine and *N, N*-dimethyltryptamine in the rat. *Biochemical Pharmacology*.

[21] Sitaram B. R., McLeod W. R. (1990). Observations on the metabolism of the psychotomimetic indolealkylamines: implications for future clinical studies. *Biological Psychiatry*.

[22] Shen H.-W., Jiang X.-L., Winter J. C., Yu A.-M. (2010). Psychedelic 5-methoxy-N,N-dimethyltryptamine: metabolism, pharmacokinetics, drug interactions, and pharmacological actions. *Current Drug Metabolism*.

[23] Yamada M., Yasuhara H. (2004). Clinical pharmacology of MAO inhibitors: safety and future. *NeuroToxicology*.

[24] Farzin D., Mansouri N. (2006). Antidepressant-like effect of harmane and other *β*-carbolines in the mouse forced swim test. *European Neuropsychopharmacology*.

[25] Litchfield J., Wilcoxon F. (1949). A simplified method of evaluating dose-effect experiments. *Journal of Pharmacology and Experimental Therapeutics*.

[26] El Hilaly J., Israili Z. H., Lyoussi B. (2004). Acute and chronic toxicological studies of Ajuga iva in experimental animals. *Journal of Ethnopharmacology*.

[27] Muñoz J. L. P., Ceinos R. M., Soengas J. L., Míguez J. M. (2009). A simple and sensitive method for determination of melatonin in plasma, bile and intestinal tissues by high performance liquid chromatography with fluorescence detection. *Journal of Chromatography B: Analytical Technologies in the Biomedical and Life Sciences*.

[28] Lowry O. H., Rosebrough N. J., Farr A. L., Randall R. J. (1951). Protein measurement with the Folin phenol reagent.. *The Journal of Biological Chemistry*.

[29] van Diermen D., Marston A., Bravo J., Reist M., Carrupt P.-A., Hostettmann K. (2009). Monoamine oxidase inhibition by *Rhodiola rosea* L. roots. *Journal of Ethnopharmacology*.

[30] dos Santos Passos C., Soldi T. C., Torres Abib R. (2013). Monoamine oxidase inhibition by monoterpene indole alkaloids and fractions obtained from *Psychotria suterella* and *Psychotria laciniata*. *Journal of Enzyme Inhibition and Medicinal Chemistry*.

